# Genetic analyses reveal complex dynamics within a marine fish management area

**DOI:** 10.1111/eva.12760

**Published:** 2019-01-20

**Authors:** Jakob Hemmer‐Hansen, Karin Hüssy, Henrik Baktoft, Bastian Huwer, Dorte Bekkevold, Holger Haslob, Jens‐Peter Herrmann, Hans‐Harald Hinrichsen, Uwe Krumme, Henrik Mosegaard, Einar Eg Nielsen, Thorsten B. H. Reusch, Marie Storr‐Paulsen, Andres Velasco, Burkhard von Dewitz, Jan Dierking, Margit Eero

**Affiliations:** ^1^ National Institute of Aquatic Resources Technical University of Denmark Silkeborg Denmark; ^2^ National Institute of Aquatic Resources Technical University of Denmark Kgs. Lyngby Denmark; ^3^ Institute of Sea Fisheries Bremerhaven Germany; ^4^ Institute of Marine Ecosystem and Fishery Science University of Hamburg Hamburg Germany; ^5^ Evolutionary Ecology of Marine Fishes GEOMAR Helmholtz Center for Ocean Research Kiel Kiel Germany; ^6^ Thünen Institute of Baltic Sea Fisheries Rostock Germany

**Keywords:** Atlantic cod (*Gadus morhua*), conservation, evolution, fisheries management, genetics, genomics, marine fishes

## Abstract

Genetic data have great potential for improving fisheries management by identifying the fundamental management units—that is, the biological populations—and their mixing. However, so far, the number of practical cases of marine fisheries management using genetics has been limited. Here, we used Atlantic cod in the Baltic Sea to demonstrate the applicability of genetics to a complex management scenario involving mixing of two genetically divergent populations. Specifically, we addressed several assumptions used in the current assessment of the two populations. Through analysis of 483 single nucleotide polymorphisms (SNPs) distributed across the Atlantic cod genome, we confirmed that a model of mechanical mixing, rather than hybridization and introgression, best explained the pattern of genetic differentiation. Thus, the fishery is best monitored as a mixed‐stock fishery. Next, we developed a targeted panel of 39 SNPs with high statistical power for identifying population of origin and analyzed more than 2,000 tissue samples collected between 2011 and 2015 as well as 260 otoliths collected in 2003/2004. These data provided high spatial resolution and allowed us to investigate geographical trends in mixing, to compare patterns for different life stages and to investigate temporal trends in mixing. We found similar geographical trends for the two time points represented by tissue and otolith samples and that a recently implemented geographical management separation of the two populations provided a relatively close match to their distributions. In contrast to the current assumption, we found that patterns of mixing differed between juveniles and adults, a signal likely linked to the different reproductive dynamics of the two populations. Collectively, our data confirm that genetics is an operational tool for complex fisheries management applications. We recommend focussing on developing population assessment models and fisheries management frameworks to capitalize fully on the additional information offered by genetically assisted fisheries monitoring.

## 1 INTRODUCTION

Genetic and genomic information is increasingly used to inform conservation and management efforts targeting natural populations (Benestan et al., 2016; Funk, McKay, Hohenlohe, & Allendorf, 2012). In marine fisheries, management units often do not match the distribution areas of biological populations (Kerr et al., 2017; Reiss, Hoarau, Dickey‐Collas, & Wolff, 2009). Increased access to genomic data is a promising development for identifying management units and connectivity patterns in species showing low levels of population structuring (Gagnaire et al., 2015; Hemmer‐Hansen, Therkildsen, & Pujolar, 2014; Kelley, Brown, Therkildsen, & Foote, 2016). Large genomic data sets facilitate the identification of genetic loci with high discriminatory power (Nielsen et al., 2012) which are useful for fast and cost‐effective identification of populations and for quantifying their relative contributions to mixed‐stock fisheries, that is, fisheries targeting more than one biological population. These developments facilitate the transfer from proof‐of‐concept to operational tools which can support and improve management in complex management scenarios. Yet, apart from classical cases such as Pacific salmonids (Dann, Habicht, Baker, & Seeb, 2013), only few examples of genetically based marine fisheries management exist (Bernatchez et al., 2017; Dahle, Johansen, Westgaard, Aglen, & Glover, 2018).

**Figure 1:**
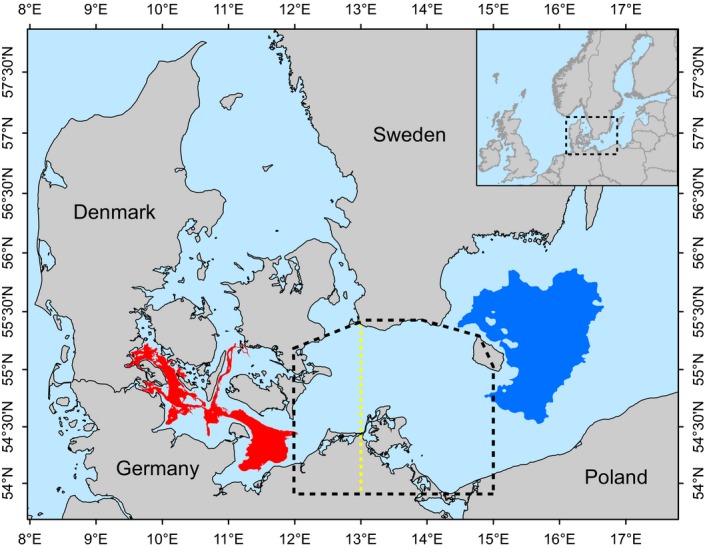
Map of the study region showing the geographical location of eastern (blue) and western (red) Baltic Sea population spawning areas used as baselines. The mixing zone is marked by a dashed black line and a dashed yellow line marks the separation of two areas currently used for stock assessment within the mixing zone. Until 2015, the mixing zone belonged exclusively to the western Baltic cod population for stock assessment and management

Atlantic cod (*Gadus morhua*) is a marine fish species of high ecological and commercial importance distributed across the northern Atlantic. It has received considerable interest from studies focusing on basic as well as applied research in both aquaculture and wild populations, and the separation of coastal and offshore populations in the Northern Atlantic (Dahle et al., 2018; Wennevik, Jørstad, Dahle, & Fevolden, 2008) represents an illustrative case of genetically informed marine fisheries management. A number of studies have identified Atlantic cod populations in the brackish water Baltic Sea as genetically unique (Berg et al., 2015; Nielsen, Hansen, Schmidt, Meldrup, & Grønkjær, 2001), presumably as a result of a combined colonization and adaptation process to the unique Baltic Sea environment (Hemmer‐Hansen et al., 2013; Nielsen, Hansen, Ruzzante, Meldrup, & Grønkjær, 2003; Nielsen et al., 2009), and possibly reflecting separation prior to the opening of the connection between the Atlantic and the Baltic Sea following the last glaciation (Sick, 1965). Adaptations to the Baltic Sea environment involve several physiological and life‐history traits such as egg buoyancy and spawning time (Hinrichsen, Hüssy, & Huwer, 2012; Nissling, Kryvi, & Vallin, 1994; Petereit, Hinrichsen, Franke, & Köster, 2014). Populations in the environmental transition zone between the North Sea and Baltic Sea have been found to be of mixed genetic ancestry (Berg et al., 2015; Nielsen et al., 2003), resembling a hybrid zone between pure North Sea and Baltic Sea populations (Nielsen et al., 2003). The major genetic break between the Baltic Sea populations is localized in the Arkona Basin region in the western part of the Baltic Sea (see Figure 1 and Nielsen et al., 2003; Pocwierz‐Kotus et al., 2015). The two cod populations show marked phenotypic differences, with eastern Baltic cod growing more slowly, maturing at a smaller size and attaining lower weight at a given length than western Baltic cod (Bagge, Thurow, Steffensen, & Bay, 1994; Berner & Vaske, 1985; Köster et al., 2017).

In terms of fisheries management, cod in the Baltic Sea is managed as two stocks, “western” and “eastern” Baltic cod, with the Arkona Basin region managed as part of the western stock until recently (Figure 1). New genetic data as well as otolith morphology and trends in biological characteristics, such as mean weight of cod (Eero, Hemmer‐Hansen, & Hüssy, 2014; Hüssy, Hinrichsen et al., 2016), have recently demonstrated that mixing of eastern and western (transition zone) cod populations occurs in the Arkona Basin region. Population mixing in this area was of considerable concern due to biased stock assessments, which were conducted separately for management areas east and west of a 15° E borderline (see Figure 1). Consequently, mixing was believed to occur within the western management area and, until recently, the assessment of the fisheries resource did not contain detailed information about the spatial and temporal scale of mixing (Eero et al., 2014). The marked life‐history differences between cod from the two biological populations in the mixing zone could act to decouple population dynamics for the two units with potential consequences for stock assessment and management. For example, differences in spawning time may result in different recruitment patterns in the two populations, and different population productivity may lead to overexploitation of the weaker population component in the mixing zone. As a result, a combination of morphological and genetic data was applied to determine the proportions of eastern and western Baltic cod in fisheries catches taken within the western management area for stock assessment purposes (Hüssy, Hinrichsen et al., 2016; ICES, 2017; Figure 1).

The stock separation used in the current management model was implemented in 2015 (Hüssy, Hinrichsen et al., 2016; ICES, 2015) and has a number of assumptions. First, it assumes that while individuals of the two populations co‐occur in the same geographical area (termed “mechanical mixing” in the following), they do not interbreed and hence a possible scenario of hybridization is disregarded. In this geographical region, early studies suggested the presence of mechanical mixing of two populations based on sample statistics of hemoglobin polymorphism data (Sick, 1965), while more recent work applying genetic markers with higher statistical power for individual‐based analyses, but also lower sample sizes, was not able to differentiate between mechanical mixing and hybridization scenarios (Nielsen et al., 2003). Although mechanical mixing was recently demonstrated with the use of highly powerful single nucleotide polymorphism genetic markers (Eero et al., 2014), the geographical extent of mixing and the possible occurrence of hybrids have so far not been rigorously assessed with tools with high statistical power. Second, the current assessment and management model only uses a coarse model of different mixing in two sub‐areas (see Figure 1) to account for the geographical variation in mixing proportions. Third, it is currently assumed that mixing proportions are similar for adults and juveniles, and similar proportions of eastern and western fish are allocated to all fish ages when performing the stock assessments. Consequently, the presence of age‐specific mixing proportions may result in biased stock assessments. Finally, so far, there is no mechanistic understanding of the mechanisms driving spatial patterns and temporal fluctuations in mixing proportions.

Here, we use genetic markers to assess the validity of these assumptions. First, we apply 483 SNP markers in a limited number of individuals and a reduced but specifically selected panel of 39 SNP markers in a large number of individuals to verify the hypothesis of mechanical mixing (i.e., the absence of hybrids) of the two populations. Subsequently, we use the reduced, so‐called high‐graded, panel of markers to study population mixing and dynamics with high spatial and temporal resolution in a large number of individuals collected within the mixing zone. We use these data to investigate if current management assumptions are accurately describing the dynamics of mixing in the region, that is, addressing the assumptions related to geographical patterns of mixing for juvenile and adults. Finally, we use the genetic data in combination with environmental data to assess possible mechanisms driving population mixing.

## 2 MATERIALS AND METHODS

### 2.1 Sampling and DNA extraction

The geographical focus of this study was at the Arkona Basin region in the Baltic Sea (Figure 1) where previous work suggested that eastern and western Baltic Sea cod populations mix. Baseline samples for the study were represented by Atlantic cod collected at spawning time in the western Baltic Sea and in the Bornholm Basin in the eastern Baltic Sea (Table 1 and Figure 1). These baselines are termed “western” and “eastern” Baltic Sea cod throughout the study. Tissue samples (gills and fins) from 2,042 individuals from the mixing zone were collected from research cruises using bottom trawling gear during a 5‐year period from 2011 to 2015 (Table 2) and stored in ethanol. For each fish, biological data such as length, weight, and maturity stage determined from gonadal maturation status was recorded. Gonadal maturation stage was used to categorize fish as either juvenile or adult and to determine whether adult fish were in spawning condition at the time of capture. All fish sampled in November 2013 and February 2014 were below 20 cm length and were therefore assumed to be juveniles. DNA was extracted from tissue samples by Chelex resin (Estoup, Largiader, Perrot, & Chourrout, 1996). In addition, we analyzed DNA extracted from 260 otoliths collected in 2003 and 2004 to examine temporal stability of population mixing (Table 3). For otoliths, we followed the procedure outlined in Bonanomi et al. (2015), including DNA extraction in a clean laboratory facility and assessment of contamination by microsatellite genotyping prior to SNP genotyping. All samples were genotyped for 39 SNP markers on a Fluidigm Biomark™ HD System.

**Table 1:** Baseline samples used for assignment and for estimating assignment power

Baseline	Sampling time	Sample size	No SNPs	Use	Source
Eastern	April, 1997	40	483/39	Assignment	Nielsen et al. (2012)
Eastern	February, 2007	40	483/39	SNP selection/Assignment	Nielsen et al. (2012)
Eastern	July/August, 2011/2012	150	39	Power estimation	This study
Western	February/March, 1996	40	483/39	Assignment	Nielsen et al. (2012)
Western	March, 2007	37	483/39	SNP selection/Assignment	Nielsen et al. (2012)
Western	February, 2012	150	39	Power estimation	This study

**Table 2:** Tissue samples analyzed from the mixing zone

Year	Month	Quarter	Total sample size	Number of spawning fish	Number of juveniles^a^	Comments
1996	February/March	1	40	0	0	Sample from Nielsen et al. (2003) re‐analyzed with 483 SNPs in this study
2011	June	2	536	152	50	
2013	July	3	41	0	41	
2013	November	4	150	0	150	All fish below 20 cm length
2014	February	1	150	0	150	All fish below 20 cm length
2014	February	1	21	19	0	Spawning fish, not used for environmental correlation
2014	April	2	289	78	19	
2014	August	3	145	0	94	
2014	October	4	229	0	51	
2015	February	1	236	2	16	
2015	July	3	90	18	23	
2015	September	3	155	0	43	

a Juveniles were identified through maturation estimation by fish dissection and by assuming that fish smaller than 20 cm were all juveniles.

**Table 3:** Otolith samples analyzed from the mixing zone. Proportions of eastern fish are shown in brackets. A Major Baltic Inflow was observed in January 2003

Year	Quarter	Western Arkona	Eastern Arkona	Total
2003	1	65 (0.28)		
	4	20 (0.25)	43 (0.93)^a^	
	Total 2003	85	43	128
2004	1	62 (0.16)		
	4	22 (0.09)	48 (0.83)	
	Total 2004	84	48	132

a Thirty‐two of the individuals were collected on a fishing trip to the westernmost part of the neighboring area, that is, to the east of the eastern border of the Arkona mixing zone. Among the 11 individuals collected within the Arkona region, the proportion of eastern fish was 0.82.

### 2.2 Development of a SNP panel for population assignment

We used a previously published data set composed of more than 1,200 SNP loci (see Nielsen et al., 2012 for details) to identify loci with high levels of population differentiation between eastern and western Baltic Sea baseline population samples collected in 2007, ranking loci based on estimates of *F*_ST_ (Weir & Cockerham, 1984). We only included SNPs within the same linkage group if the correlation between markers was very low (*r* ^2^ < 0.01). In addition, markers on different linkage groups were also only selected if they showed low levels of linkage disequilibrium (max *r* ^2^ < 0.25) to ensure that all markers would provide independent information. This procedure also limited biases for population assignment from including linked markers in the well‐described highly differentiated genomic regions (at least one corresponding to major chromosomal inversions, Kirubakaran et al., 2016) in the cod genome, some of which show high levels of differentiation for the Baltic Sea and transition zone populations (Berg et al., 2015). Baseline samples collected in 1996/1997 and 2007 were not significantly different and were pooled as assignment baselines to get a more complete representation of genetic variation in the two populations. Assignment to the most likely baseline (eastern or western Baltic Sea) was based on genotype likelihoods (following Rannala & Mountain, 1997), which were used to calculate assignment scores in the programme GeneClass2 (Piry et al., 2004). The assignment score is the ratio of the maximum genotype likelihood to the sum of all likelihoods (here the sum of the likelihoods in the two possible baseline samples). Individuals were assigned to the baseline with the highest score. Statistical power for assignment was evaluated in baseline samples collected in 2012 by self‐assignment using the leave‐one‐out procedure. By estimating power in a different sample (“hold‐out sample”) than the one used for locus selection, we avoided common problems of “high grading bias” (Anderson, 2010). Assignment power was evaluated by estimating the number of misassigned individuals in the baseline samples and by examining the distribution of likelihood ratios, following Ogden and Linacre (2015).

### 2.3 Distinguishing between hybridization and mechanical mixing

To attain high statistical power for distinguishing between hybrids and pure parental genotypes, we re‐analyzed the Arkona Basin sample collected in 1996 from Nielsen et al. (2003) with the use of SNP markers (Table 2; see Nielsen et al., 2012 and Supporting Information Table S1 for details of the data set). In the original publication (Nielsen et al., 2003), it was not possible to distinguish between the two scenarios with the use of nine microsatellite markers. Here, we selected SNPs to be distributed throughout the cod genome by selecting markers with a minimum distance of 1 cM on the cod linkage map (Borza, Higgins, Simpson, & Bowman, 2010; Hubert, Higgins, Borza, & Bowman, 2010). This procedure resulted in a data set consisting of 483 markers. We used the western and eastern Baltic Sea baseline samples collected in 1996/1997 and 2007 as parental populations (Table 1) and used R (R Core Team, 2016) to simulate pure parental and F1 hybrid individuals by drawing alleles with probabilities equal to baseline sample allele frequencies. The simulated individuals were analyzed with the Arkona Basin sample in the model‐based clustering programme STRUCTURE (Hubisz, Falush, Stephens, & Pritchard, 2009) using prior population information for the parental populations. Individual admixture coefficients and their 95% confidence intervals (CIs) were compared for simulated and Arkona Basin individuals. Due to limited statistical power with a reduced number of markers, the same approach could not be used for the majority of individuals in this study as they were only genotyped for 39 SNPs. However, the large number of fish analyzed permitted an examination of the distribution of assignment scores which could still be used for estimating the extent of hybridization. Large numbers of fish with intermediate assignment scores would indicate frequent hybridization among the analyzed individuals.

### 2.4 Modelling the geographical distribution of mixing

We used binomial generalized linear models (GLM) to examine geographical patterns of mixing for juveniles and adults and the relationships between mixing proportions and potential environmental drivers of mixing. Specifically, we modelled the distribution of mixing proportions while accounting for spatial correlation induced by the sampling design using the following base model extended to include geographical and/or age‐class (juvenile vs. adult) covariates according to Table 4:

$${\text{East}}_{\mathrm{ip}}∼\text{Binomial(}\pi_{\mathrm{ip}},N_{\mathrm{ip}})$$

**Table 4:** Binomial generalized linear models compared for fits to observed mixing proportions in the Arkona Basin region

Model	Main effects	Geographic and age‐class covariates^a^	WAIC	ΔWAIC^b^
M0	O2+sal+temp	(none)	620.27	98.28
M1	O2+sal+temp	Area	614.65	92.66
M2	O2+sal+temp	Juvenile	549.21	27.22
M3	O2+sal+temp	*F*(utmX)	615.81	93.8
M4	O2+sal+temp	*F*_juvenile_(utmX)	521.99	—

a “Area” indicates the area definitions currently used for stock assessment (see Figure 1). “*F*(utmX)” is the model including a longitudinal smoother. “Juvenile” indicates models taking variation between juveniles and adults into account.

b Difference between best model (M4) and current model.

$$E\left({\text{East}}_{\mathrm{ip}}\right)=\pi_{\mathrm{ip}}\times N_{\mathrm{ip}}$$

$$\text{var}({\text{East)}}_{\mathrm{ip}}\;\text{=}\;\pi_{\mathrm{ip}}\times N_{\mathrm{ip}}\times \left(1-\pi_{\mathrm{ip}}\right)$$

$$\text{logit}\left(\pi_{\mathrm{ip}}\right)={\text{salinity}}_{\mathrm{ip}}{\;\text{+ oxygen}}_{\mathrm{ip}}{\;\text{+ temperature}}_{\mathrm{ip}}\;\text{+}\;u_{\mathrm{ip}}$$

$$u_{\mathrm{ip}}∼\text{GMRF}\left(0,\;\sum_{p}\right)$$

In these models, number of eastern fish, East*_ip_*, out of *N_ip_* fish from sampling event (i.e., trawl haul) *i* at sampling period (defined as year‐quarter combinations) *p* is assumed to follow a binomial distribution with probability *π*_ip_. Expected values and variance of East*_ip_* are defined by *E*(East*_ip_*) and var(East*_ip_*). Salinity, oxygen content, and water temperature in the Arkona Basin at the time and location of catch were extracted from the hydrodynamic Kiel Baltic Sea Ice‐Ocean Model (BSIOM, Lehmann & Hinrichsen, 2000 and Lehmann, Krauß, & Hinrichsen, 2002; see Supporting Information Appendix S1 for details) and included as main effects salinity*_ip_*, oxygen*_ip_*, temperature*_ip_* in the linear predictor function. These continuous covariates were standardized using *x*_std_ = (*x* − mean(*x*))/*SD*(*x*) prior to modelling.

To assess assumptions currently used for management, we fitted five models (Table 4). M0 is the base model as described above and functions as a reference model. Current management assumes that a split into two sub‐areas (west and east of 13° E; see Figure 1) captures the geographical variation in mixing proportions and that proportions are similar for adults and juveniles. These assumptions are evaluated in models M1 and M2, respectively. M1 includes factor “Area”, describing whether observation *ip* is from the area west or east of 13° E, whereas M2 includes factor “Juvenile”, describing whether observation *ip* pertains to juvenile or adult fish. Due to sparse number of samples containing adults from the western area, the interaction between *Area* and *Juvenile* could not be tested. In M3, a second‐order random walk model “*F*(utmX*_ip_*)” was included to capture an overall (potentially non‐linear) east–west trend on *π_ip_* as the study area extension was primarily in the east–west direction. This was extended further in M4 by allowing the second‐order random walk to vary between juveniles and adults. Models were compared using the Watanabe–Akaike information criteria (WAIC; Zuur, Ieno, & Saveliev, 2017). The term *u_ip_* is a random intercept assumed to be spatially correlated with mean 0 and sampling period‐specific covariance matrix *∑_p_*, that is, a replicated spatial random field that is allowed to differ between sampling periods *p*. *u_ip_* is assumed to be Markovian and thus follow a Gaussian Markov random field (GMRF) and the associated covariance matrices are modelled using Matérn correlation function and numerically approximated using SPDE (continuous domain stochastic partial differential equation). A logit link function was used to model *π_ip_* as a function of the linear predictor function. The model was fitted in a Bayesian framework using R (R Core Team, 2016) and the package R‐INLA (Lindgren, Rue, & Lindstrom, 2011; Rue, Martino, & Chopin, 2009) following Zuur et al. (2017). Diffuse priors were used for all parameters. Model validation and diagnostics were performed using Pearson residuals on population level (i.e., excluding random effects) following procedures as described in Zuur et al. (2017). Only data from stations within the mixing zone in Figure 1 were included in the GLM model. Additionally, data sampled prior to 2011 were excluded as the geographic resolution of these was inadequate for this model, and data from two stations were excluded as environmental covariates were unavailable for these. It should be noted that attempts to model a system as complex and dynamic as the mixing of Baltic Sea populations using simple predictors as done here inevitably will be an oversimplification. However, we believe that the present data set does not warrant further complexity in the models considering the available sample sizes.

Our sampling design also allowed a more qualitative assessment of potential effects of major environmental changes observed in January 2003 and December 2014, where strong inflow of high salinity water to the Baltic Sea was observed (Major Baltic Inflow event, Mohrholz, Naumann, Nausch, Krüger, & Gräwe, 2015), by comparing the geographical patterns of mixing before and after inflows.

## 3 RESULTS

### 3.1 Development of a SNP panel for population assignment

We identified 39 SNP markers which provided high statistical power for population assignment to eastern and western Baltic cod populations (see Supporting Information Table S2 for detailed information). Because of the different selection criteria used for the two SNP panels, only 23 of the markers in this panel overlapped with markers in the 483 SNP data set used to differentiate between hybridization and mechanical mixing below (see Supporting Information Tables S1 and S2). For the panel of 39 SNPs, only one individual from the western Baltic Sea baseline was assigned to the eastern Baltic Sea baseline, corresponding to a misassignment rate of ~0.67%. No individuals were misassigned from the eastern Baltic baseline to the western Baltic Sea baseline. Furthermore, the distributions of likelihood ratios were well separated, demonstrating that assignment was unambiguous for the majority of baseline fish (Supporting Information Figure S1). The “average” fish in the western Baltic baseline was more than 22 million times more likely to assign to west than to east, while the “average” fish in the eastern Baltic baseline was more than 2 million times more likely to assign to east than to west.

### 3.2 Distinguishing between hybridization and mechanical mixing

**Figure 2:**
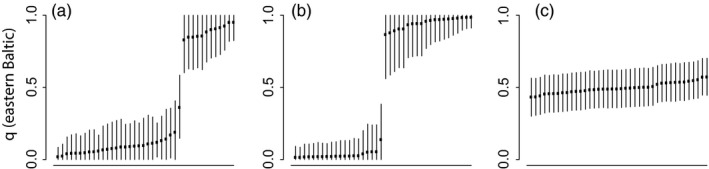
Individual admixture proportions with 95% confidence intervals in the sample from the Arkona Basin analyzed with 483 SNPs in (a), simulated pure parental individuals in (b) and simulated F1 hybrids in (c). Pure eastern Baltic fish have an admixture coefficient close to 1. Data in (b) were simulated assuming a 50/50 mixing ratio

For the 483 SNP data set, the estimation of individual admixture coefficients showed that 95% CIs for simulated parental individuals always overlapped with either 0 or 1 (with 1 corresponding to pure eastern and 0 to pure western origin) but never both. Simulated F1 hybrids had intermediate admixture coefficients and 95% CIs which never included 0 or 1 (Figure 2b,c). The admixture coefficients for the 40 Arkona Basin individuals almost exclusively overlapped with either 0 or 1 (Figure 2a), thus supporting that they represented genotypes with pure parental origins. Only one individual showed an intermediate admixture coefficient with 95% CI not overlapping with 0 or 1. For the 39 SNP data set with 2,270 successfully genotyped individuals collected from the mixing zone, only 28 individuals (~1%) had assignment scores below 90 (Supporting Information Figure S2), suggesting the presence of very few individuals of admixed genetic ancestry since a value close to 50 would be expected for F1 hybrids.

### 3.3 Assignment of fish from the mixing zone

**Figure 3:**
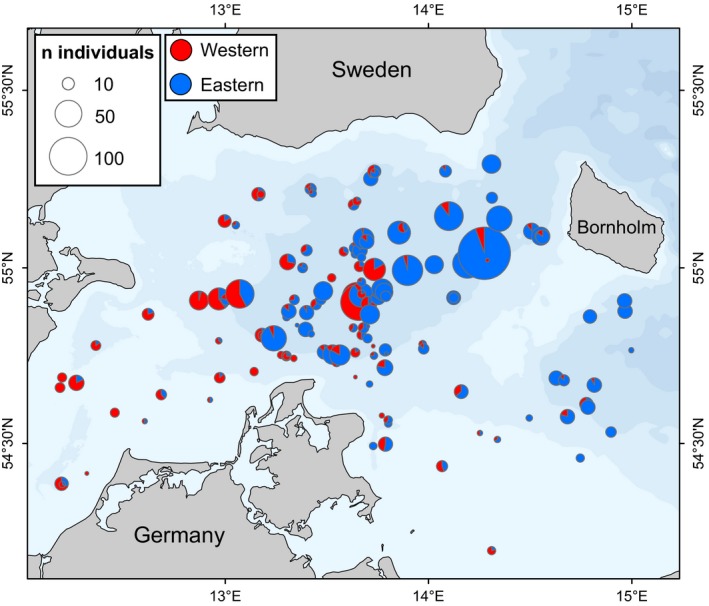
Proportions of eastern and western cod for all juvenile and adult samples collected from 2011 to 2015

**Figure 4:**
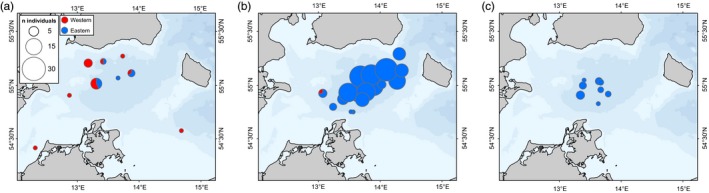
Proportion of eastern and western cod among spawning fish collected in quarter 1 (*n* = 21) in (a), quarter 2 (*n* = 230) in (b) and quarter 3 (*n* = 18) in (c) in samples collected from 2011 to 2015

**Figure 5:**
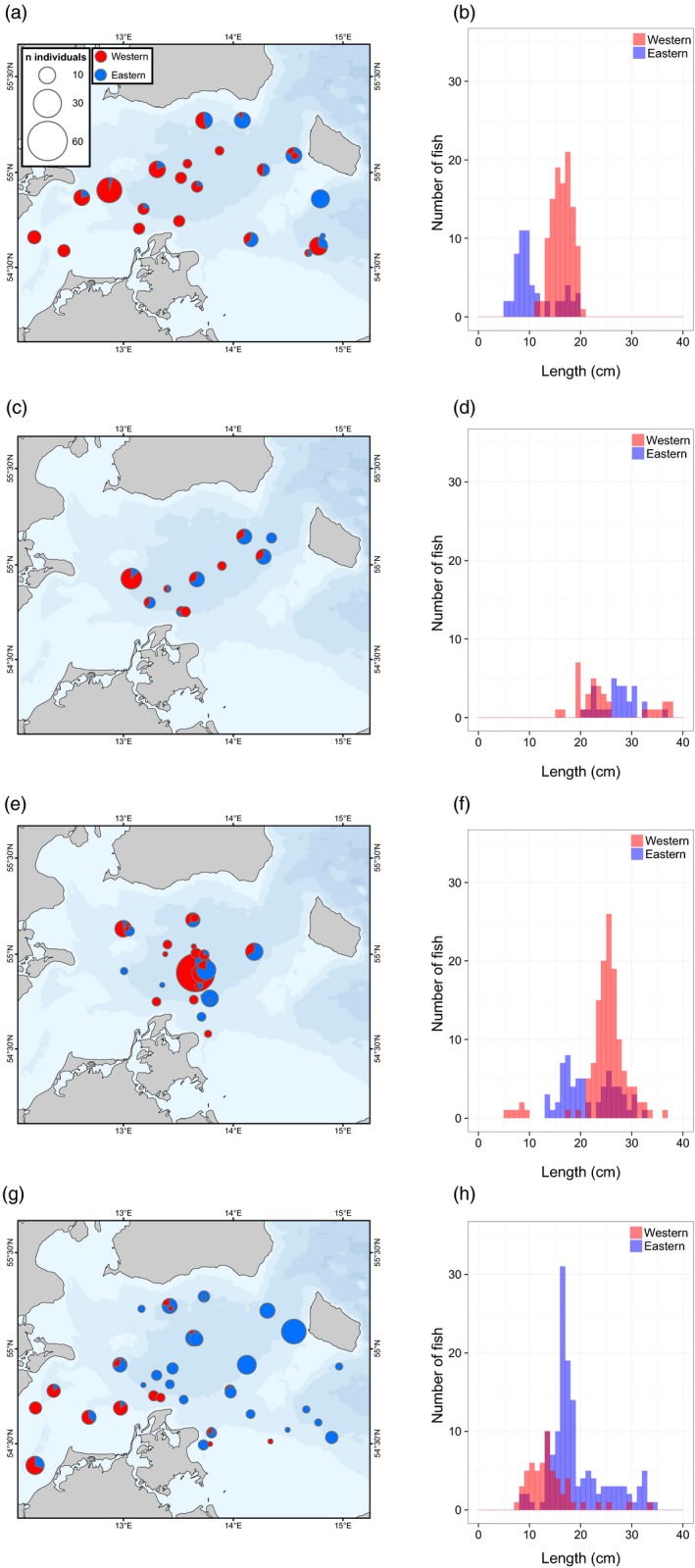
Spatial distributions of mixing proportions and size distributions of juvenile cod for quarters 1 (a and b, *n* = 166), 2 (c and d, *n* = 66), 3 (e and f, *n* = 195) and 4 (g and h, *n* = 200). Overlapping size distributions indicated by shading of colors. Data were combined across years

Assignment of cod collected between 2011 and 2015 showed mechanical mixing of eastern and western cod in the study region (Figure 3). Analyses of samples from 1996, 2003, and 2004 confirmed the presence of both populations also in these years (Figure 2 and Table 3). Furthermore, there was a geographical gradient from east to west with higher proportions of eastern fish in the collections in the central and eastern parts of the area (Figure 3 and Table 3). In the first quarter of the year, fish in spawning condition represented both western and eastern cod, whereas collections of second and third quarter spawning fish were almost exclusively of eastern origin (Figure 4). Analyses of the origins of juveniles showed that mixing patterns were highly dynamic (Figure 5 for data pooled across years). For example, a difference in geographical patterns was evident for juveniles, where western fish dominated the central parts of the mixing zone in quarters 1–3 while eastern fish were more frequent in the fourth quarter of the year (Figure 5). The size distributions of the juvenile fish from the two populations were also markedly different (Figure 5), showing clear patterns of discrete year‐classes of eastern and western fish at the different times of the year. It should be noted that data were pooled across years to increase total sample size, that sample size was limited for the second quarter and that the first quarter was dominated by sampling fish <20 cm. Thus, it is likely that we have missed larger juveniles in the first quarter of the year. Still, the bimodal distributions for both eastern and western juveniles observed in some of the quarters indicated that collections included at least two year‐classes of juveniles from the populations (Figure 5).

### 3.4 Modelling the geographical distribution of mixing

**Figure 6:**
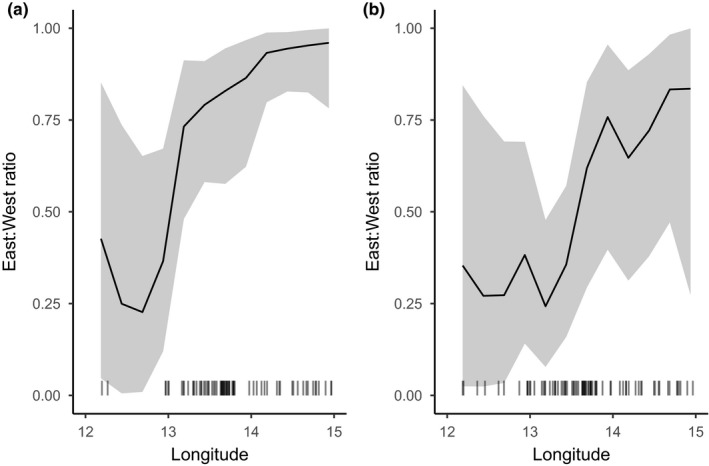
Modelled geographical distribution of mixing proportions for adults (a) and juveniles (b) for model M4 conditional on oxygen, salinity and temperature being fixed at mean values (see Supporting Information Figure S3). Shaded area describes 95% credible intervals and the geographical (longitudinal) location of sampling stations is shown above the *x*‐axis. Note the low geographical coverage for adults in the western part of the study region, which prevented a detailed modelling of the effects of Area and Juvenile

**Figure 7:**
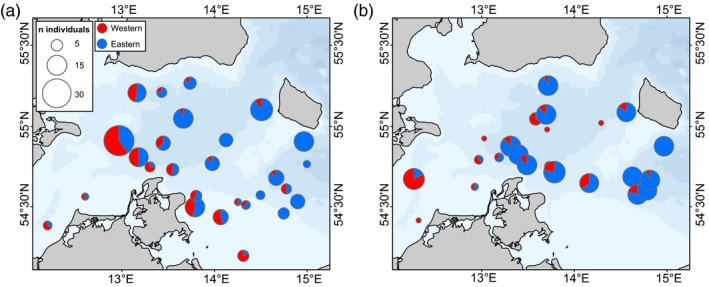
Proportions of eastern and western cod before (*n* = 229) in (a) and after (*n* = 236) in (b) a Major Baltic Inflow of high salinity water in December 2014

Model comparisons showed the best performance of the model including information about life stage (juvenile vs. adult) and using a gradual geographical transition in mixing proportions (M4; Table 4). This model showed a clear effect of longitude and life stage, as illustrated by the modelled geographical distributions of mixing proportions for the two life stages separately (Figure 6). Although 95% credible intervals were wide, the model suggested that the longitude effect was non‐linear (Figure 6), and both juveniles and adults showed major geographical shifts from high proportions of eastern fish in the eastern parts to low proportions in the western parts of the study area. A rapid transition from predominantly western to eastern Baltic cod occurred at a longitude around 13° E for adults (Figure 6a) and around 13.5° E for juveniles (Figure 6b). It should, however, be noted that our sampling coverage for adults in the westernmost parts of the study area was relatively poor. When life stage was not taken into account, the model using the area definitions currently used for stock assessment (M1) performed clearly better than a model without geographical information (M0) and similarly to a model allowing a gradient in mixing proportions (M3). None of the models showed significant effects of the environmental variables (O_2_, salinity, and temperature) as the 95% credible intervals for parameter estimates included 0 (Supporting Information Figure S3). There was no clear increase in proportions of the high salinity adapted western Baltic cod population associated with Major Baltic Inflow events in January 2003 and December 2014 (Figure 7 and Table 3).

## 4 DISCUSSION

Sustainable fisheries management relies critically on our ability to identify biological populations, and to incorporate information about their spatial distributions and mixing into management (Bernatchez et al., 2017; Heath et al., 2014). Genetic markers specifically selected to be informative for population identification, so‐called high‐graded marker panels, have been shown to provide efficient traceability tools for marine fisheries management (Grewe et al., 2015; Montes et al., 2017; Nielsen et al., 2012). This approach consists of a two‐step procedure; an initial screening of genomes for signatures of population divergence and a subsequent filtering and genotyping of the markers carrying strong signals of divergence. Highly divergent genomic regions may be related to adaptive population divergence in response to environmental differences or linked to cryptic population structure, and may not be observed through the use of a random selection of genetic markers (Gagnaire et al., 2015). Consequently, such markers may serve as efficient population tags that can be used to identify the population of origin of individuals. However, since the markers may be located in genomic regions affected by selection, it is generally recommended that baselines should be re‐evaluated at regular intervals to assess temporal stability of genetic signatures, in particular in situations under dynamic environmental conditions (Nielsen et al., 2012).

The data presented in this paper provide high spatial and temporal resolution of population interaction of Atlantic cod populations in the western Baltic Sea. In particular, the combination of high genome coverage in a limited number of samples and data from a high‐graded SNP panel genotyped in thousands of individuals was very useful for investigating complex population dynamics including a direct assessment of assumptions currently used in fisheries management. The high‐graded SNP panel was specifically tailored to the study questions and provided very high statistical power for identifying the Atlantic cod populations expected to co‐occur in the study region. As such, our study also serves as an example of an application of a minimum marker panel with maximum statistical power in a marine fisheries management case (Nielsen et al., 2012).

### 4.1 Assumption: Mechanical mixing is the main form of interaction between populations

In this study, we have confirmed that eastern and western Baltic cod co‐occur in the contact zone between eastern and western Baltic Sea cod populations, and that mechanical mixing and not hybridization is the major form of interaction between these populations. Thus, our results support the first important assumption under the current management scheme, and align with an increasing number of mixed‐stock scenarios recently unveiled in marine fishes with the use of genetic data, for example, Atlantic herring (Bekkevold et al., 2015, 2011) and cod in the northern Atlantic (Bonanomi et al., 2015; Johansen et al., 2018; Therkildsen et al., 2013). Our results also align with early work inferring mechanical mixing in the Arkona Basin region based on sample departures from Hardy–Weinberg expectations in hemoglobin polymorphism data (Sick, 1965). More powerful genetic markers should provide resolution at the individual fish level; however, previous work was not able to determine the most likely scenario in this geographical area due to a lack of statistical power with the genetic methods applied at the time and a limited sample size in the contact zone between the two populations (9 microsatellite loci and 59 individuals; Nielsen et al., 2003). With 483 SNP markers, we were able to move from inferences based on sample summary statistics to analyses on the individual level and hence to differentiate between pure parental and F1 hybrid genotypes, demonstrating the increased power attained with genomic tools in shallow structure scenarios (Hemmer‐Hansen et al., 2014). Although our 39 SNP panel did not allow unambiguous differentiation between parental and hybrid genotypes at the individual fish level, we found very few intermediate assignment scores among more than 2,000 fish analyzed with this marker set. Thus, these data support the results from the 483 SNP analyses, which had higher statistical power for analysis of individual fish but were conducted on a much smaller number of fish. This raises the question of how this type of mixing can be maintained over the years without resulting in hybridization and a break‐down of population structure (Taylor et al., 2006). Despite overlapping geographical distributions, western and eastern Baltic cod have temporally distinct spawning times. While western Baltic cod spawning is restricted to a few weeks in early spring, eastern Baltic cod spawn over a prolonged period of time peaking in the summer months (Hüssy, 2011; Figure 4). Additionally, egg buoyancy differs between the two populations, where the eggs of the western stock require much higher salinities to remain buoyant than those of the eastern stock (Nissling & Westin, 1997; Petereit et al., 2014). The environmental conditions are rarely supporting survival of western cod eggs in the Arkona Basin region, the main limiting factors being low temperature during the spawning time of western cod (Köster et al., 2017) and sedimentation due to drift toward the east (Hinrichsen et al., 2012; Petereit et al., 2014). During the 2000s, the environmental conditions for reproduction in the Arkona Basin were generally more favorable for eastern than for western Baltic cod. Finally, it is possible that the substantial genomic differentiation between eastern and western Baltic Sea cod populations (Pocwierz‐Kotus et al., 2015) is also involving genomic incompatibilities (Bierne, Welch, Loire, Bonhomme, & David, 2011) which are not directly linked to adaptation to the specific environments inhabited by the two populations but still prevent hybridization between them. These mechanisms may be sufficient to prevent extensive hybridization between the two populations. Mechanical mixing in the contact zone in the Arkona Basin region may seem contradictory to previous work which has suggested that the entire geographic transition zone between the North Sea and Baltic Sea is best described as a hybrid zone between pure North Sea and Baltic Sea populations (Nielsen et al., 2003). However, it is possible that occasional hybridization events, for example, in the short period in early spring were both populations spawn in the region (Figure 4) or under certain environmental conditions in this highly environmentally dynamic region (Mohrholz et al., 2015), may generate pulses of gene flow which would result in a hybrid zone signature. In fact, we did observe one individual with an intermediate admixture coefficient and 95% CI non‐overlapping with 0 or 1, suggesting that hybridization does occur.

### 4.2 Assumption: Geographical patterns of mixing are accurately described by two sub‐areas in the mixing zone

The data confirmed the east–west gradient with respect to the proportion of eastern fish in the contact zone (Hüssy, Hinrichsen et al., 2016). The spatial modelling of the data showed that the co‐occurrence is best explained by a gradient and by taking life stage (juvenile vs. adult) into account (model M4). Without life stage information, both the Area model (M1) and the model describing the gradual geographic change (M3) provided better fits than a model without geographical information (M0). In fact, the Area model performed slightly better, and the geographical trends in the data under the best model suggest that the sub‐areas currently used for stock assessment (split at 13° E; ICES, 2017) describe the geographical trends in these data quite well, at least for the individuals analyzed in this study. However, our data also suggest that the current stock assessment approach does not capture the full geographic complexity of mixing (see next section). It should be noted that we could not evaluate models including temporal variation (within and between years) due to limited data, and temporal variation in mixing may thus be found within the region. Seasonal variation in mixing proportions has been observed for Atlantic herring (Bekkevold et al., 2011), and also in Atlantic cod in the current study region (Hüssy, Hinrichsen et al., 2016), although no consistent pattern of variation was observed between years in the latter study.

Our data from archived otoliths collected in 2003 and 2004 indicate that mixing is not a recent phenomenon. This implies that eastern fish were also present in the area at times with much smaller population sizes of eastern Baltic cod than estimated for late 2000s to early 2010s (Eero et al., 2014), as also suggested by morphological data collected from otoliths (Hüssy, Hinrichsen et al., 2016). Our data further suggested the presence of a strong east–west gradient in mixing proportions in samples from 2003 to 2004, similarly to the pattern observed in contemporary data collected after 2011, although it should be noted that the samples represent much lower geographical resolution than the contemporary data set. Notably, the time series of samples represented a period including dramatic environmental fluctuation in the form of inflows of high saline water to the Baltic Sea (Major Baltic Inflows, MBIs, Mohrholz et al., 2015). Particularly strong MBIs, leading to more saline conditions extending further eastwards in the Arkona Basin, were observed in January 2003 and December 2014 but were not associated with dramatically higher proportions of the presumably high salinity adapted western Baltic population after the inflow (Table 3 and Figure 7). Indeed, statistical modelling of the full contemporary data set showed that none of the examined environmental parameters within the mixing zone (salinity, temperature, and oxygen) were associated with the variation in mixing proportions. This suggests that mixing may be driven by population‐specific dynamics in the two independent populations, and therefore perhaps environmental conditions outside the geographical region studied here, rather than variation in environmental conditions within the Arkona region (see also discussion on juveniles below).

### 4.3 Assumption: Mixing proportions are similar for juveniles and adults

Our data clearly showed a better fit to models when they included information on life stage. Thus, although the two life stages showed similar overall geographic trends (i.e., an east–west gradient), the results also indicated that the major geographical break in mixing proportions may be different for juveniles and adults, at least for the data included in this study. Consequently, the result indicates that the current approach assuming similar proportions for juveniles and adults does not accurately account for the dynamics of mixing, and that proportions should therefore be estimated for both life stages separately to fully track the dynamics of mixing in the region.

The geographical distributions and size distributions of small juveniles from the two populations provide interesting information about the underlying dynamics potentially driving juvenile mixing proportions, and they indicate important effects of differences in reproductive biology and growth in the two populations (Hüssy, 2011). The consequences are directly observed as differences in the length distributions of the fish from the two populations (Figure 5 for data combined across years). In the first quarter, we observed two eastern and one western year‐class, most likely corresponding to eastern fish born in the previous year's summer (6 months old) and in the summer the year before (18 months old) and western fish born in the previous years’ spring (12 months old). These three year‐classes are also seen in the third quarter samples, along with the indication of the young‐of‐the‐year western cod (<10 cm long) born in the spring of the same year. Here, the eastern year‐classes would be 1 and 2 years old, respectively, suggesting that not all eastern fish have attained sexual maturity (i.e., recruited to the adult population) during their second summer. In the fourth quarter, the young western cod were still sampled, but the dominating western year‐class from the third quarter was no longer present, indicating that these fish attain sexual maturity during their second winter at an age of 2. Although we interpret the absence of juveniles of specific ages as evidence of sexual maturation and recruitment to the adult population, it should also be noted that these patterns may be affected by migration to shallow water which are not easily accessed by sampling vessels. Furthermore, sampling was heterogeneous across years and the pooled data across years may therefore have been affected by differences in year‐class strengths between years. Still, the data indicate short‐term intra‐annual variation associated with the recruitment dynamics of the two populations and that the proportions of mixing of juveniles vary with differences in year‐class strength of the populations having different spawning times. Consequently, seasonal differences in mixing proportions may occur. Although spawning of fish from both populations does seem to occur within the geographical regions studied here (Figure 4), the strengths of the year‐classes from the different populations may also be related to conditions in the primary spawning areas for the two populations, which are located outside the mixing zone (Figure 1). Disentangling population‐specific sexual maturation and recruitment to the adult populations is critical for assessment and management purposes. Thus, a greater focus on population‐specific dynamics of juveniles versus adults is recommended to provide more accurate estimates of the stock status and management advice in this complex mixing area.

Earlier work has used simulations to evaluate possible consequences of disregarding independent population dynamics of identified genetic units within a management area (Goethel & Berger, 2017; Heath et al., 2014). Typically, the main concern is overexploitation of weaker components if spatial sub‐structuring is disregarded (Bonanomi et al., 2015; Goethel & Berger, 2017; Kerr et al., 2017; Reiss et al., 2009). Here, we confirm that the two biological units indeed follow unique growth and maturation trajectories on short‐term spatial and temporal scales, resulting in independent recruitment of juveniles to the two adult populations. Importantly, since the size distributions of the two populations overlap, it is not possible to track these cohorts through simply examining size distributions. Previous work has used otolith‐based estimation of individual juvenile time of birth to identify the population of origin (Oeberst & Böttcher, 1998). However, such analyses are relatively time‐consuming, and, as we have also shown in our study, spawning time of the two populations may be partly overlapping. Consequently, genetic assignment may be an efficient tool for tracking population‐specific recruitment patterns in future work.

## 5 CONCLUSIONS

We have shown here that knowledge of population origin can provide an increased understanding of complex dynamics in a marine fishery on mixed populations. We evaluated the validity of current assumptions used in stock assessment of mixing Baltic cod stocks. Our analyses contribute to refining future assessment and management procedures; in particular, in relation to differentiating mixing patterns for juveniles and adults and to examining temporal patterns of mixing with relatively high geographical resolution. Here, genetics provide a fast and operational tool applicable to all life stages and even historical samples (Bonanomi et al., 2015). Currently, otolith‐based shape analyses are regarded a cheaper alternative to genetics for population identification of cod in the western Baltic Sea, but this method requires continuous ground‐truthing against a genetic baseline because otolith shapes are likely affected by complex interactions between environment and genetic background (Hüssy, Mosegaard et al., 2016). As recent developments in genotyping technology applications promise major reductions in analysis cost per individual (Aykanat, Lindqvist, Pritchard, & Primmer, 2016; Campbell, Harmon, & Narum, 2015), it is likely that genetics may be a primary tool for future marine fisheries management and monitoring (Bernatchez et al., 2017), as currently implemented on a routine basis for Pacific salmonids (Dann et al., 2013) and Atlantic cod in the northern Atlantic (Dahle et al., 2018). Our study highlights the Baltic Sea cod as a relevant case where genetic tools are already operational. In addition, genetic analyses could be applied to egg and larval stages to examine spawning dynamics and early life stage mortality of the different populations. These data would be important for estimating the contribution of spawning within the mixing zone to the productivity of the two populations.

Several marine fish species show similar complex patterns of population interaction as the Baltic cod examined in this study (Bekkevold et al., 2015; Dahle et al., 2018; Johansen et al., 2018; Kerr et al., 2017; Reiss et al., 2009; Saha et al., 2017; Wennevik et al., 2008). As genomic resolution continues to increase in species of interest to fisheries management, it is likely that high powered genetic tools will soon be operational for a range of species. To capitalize fully on this promising development, concomitant advances in stock assessment methods and management frameworks that take complex mixing dynamics into account will be needed.

## CONFLICT OF INTEREST

None declared.

## DATA ARCHIVING STATEMENT

Data available from the Dryad Digital Repository: https://doi.org/10.5061/dryad.83765sm
