# Supplementary material for: Genetic analyses reveal complex dynamics within a marine fish management area
Source: Evol Appl. 2019 Jan 20;12(4):830–44. doi: 10.1111/eva.12760 (PMC6439499; doi:10.1111/eva.12760)
Supplement: Supplementary file 1 [file EVA-12-830-s001.pdf]

Supporting information for: Genetic analyses reveal complex dynamics within a marine fish management area

Appendix S1. Detailed information about environmental parameters included in generalized linear modelling

The abiotic factors S,T and O_2 at the sample locations during sampling efforts were taken from the hydrodynamic Kiel Baltic Sea Ice-Ocean Model (BSIOM, Lehmann & Hinrichsen, 2000; Lehmann et al., 2002). The horizontal resolution of the coupled sea ice–ocean model is at present 2.5 km. In the vertical 60 levels are specified, which enables the upper 100 m to be resolved into levels of 3 m thickness. The model domain comprises the Baltic Sea, Kattegat and Skagerrak. The oxygen conditions in the entire Baltic Sea are described by an oxygen consumption sub-model coupled to BSIOM (Lehmann et al., 2014). At the western boundary, a simplified model of the North Sea is connected to the Baltic model domain to provide characteristic North Sea water masses entering the domain. The model is forced by low frequency sea level variations in the North Sea/Skagerrak region calculated from the BSI (Baltic Sea Index, Lehmann et al., 2002; Novotny et al., 2006), and by ERA-Interim reanalysis fields (Dee et al., 2011). These forcing data sources allow for a simulation of the period since 1979 and are provided in regular updates until the present. The model setup was run for the period 1970—2015. Hydrographic property fields of temperature, salinity and oxygen are extracted in daily resolution and averaged into monthly data grids of the entire modeling domain.

For samples taken in 2003 and 2004 abiotic conditions at the bottom of the center points of the ICES statistical rectangles 38G2 and 38G4 were extracted from the modeled property fields of the corresponding months and used as best approximation of the habitat characteristics accompanying these samples. The contemporary sample stations starting in 2011 could be assigned to more specific locations. For these samples the modeled S, T and O_2 values at the sea floor of the grid point in closest proximity was extracted from the property fields and assigned to the genetic samples.

Reverences

Dee, D.P., S. M. Uppala, A. J. Simmons , P. Berrisford , P. Poli, S. Kobayashi , U. Andrae , M. A. Balmaseda, G. Balsamo, P. Bauer, P. Bechtold , A. C. M. Beljaars, L. van de Berg, J. Bidlot, N. Bormann, C. Delsol, R. Dragani , M. Fuentes, A. J. Geer , L. Haimberger , S. B. Healy, H. Hersbach, E. V. Holm , L. Isaksen, P. Kallberg , M. Köhlera , M. Matricardi, A. P. McNally, B. M. Monge-Sanz, J.-J. Morcrette, B.-K. Park, C. Peubey, P. de Rosnay, C. Tavolato, J.-N. Thepauta and F. Vitart, 2011. The ERA-Interim reanalysis:

configuration and performance of the data assimilation system. Q. J. R. Meteorol. Soc. 137: 553–597, April 2011 A

Lehmann, A., Hinrichsen, H.-H., 2000. On the thermohaline variability of the Baltic Sea. Journal of Marine Systems, 25: 333-357.

Lehmann, A., Krauß, W., Hinrichsen, H.-H., 2002. Effects of remote and local atmospheric forcing on circulation and upwelling in the Baltic Sea. Tellus, 54A: 299–316.

Lehmann, A., Hinrichsen, H. H., Getzlaff, K. ,Myrberg, K., 2014. Quantifying the heterogeneity of hypoxic and anoxic areas in the Baltic Sea by a simplified coupled hydrodynamic-oxygen consumption model approach. Journal of Marine Systems, 134: 20–28.

Novotny, K., Liebsch, G., Lehmann, A., Dietrich, R., 2006. Variability of sea surface heights in the Baltic Sea: An intercomparison of observations and model simulations, Marine Geodesy, 29, 113-134.
