# Supplementary material for: Genetic analyses reveal complex dynamics within a marine fish management area
Source: Evol Appl. 2019 Jan 20;12(4):830–44. doi: 10.1111/eva.12760 (PMC6439499; doi:10.1111/eva.12760)
Supplement: Supplementary file 4 [file EVA-12-830-s004.pdf]

Supporting information for: Genetic analyses reveal complex dynamics within a marine fish management area

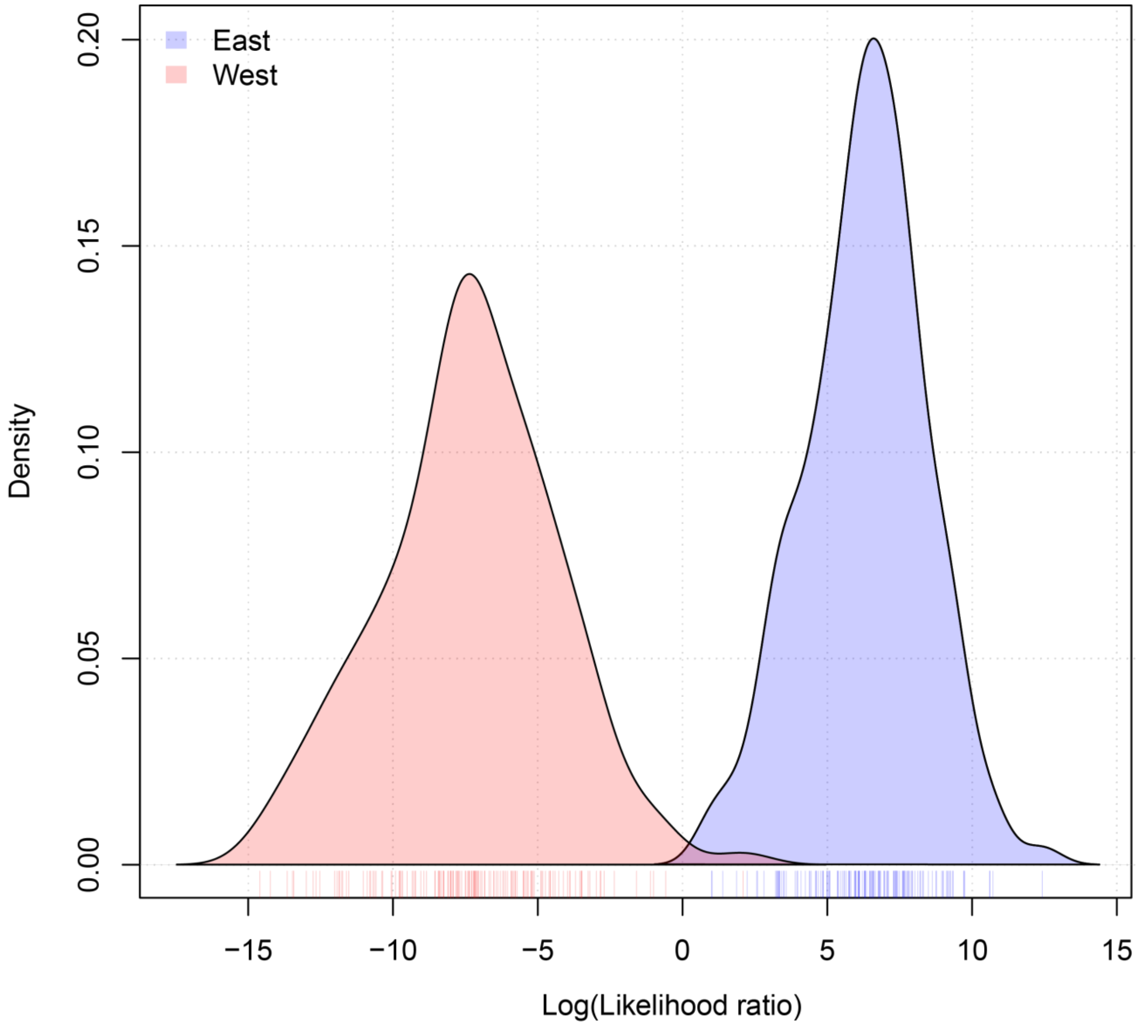

Figure S1. Distributions of Log(Likelihood ratios) in 150 eastern and 150 western baseline samples collected in 2011/2012 based on the 39 SNP assignment panel
