# Supplementary material for: Genetic analyses reveal complex dynamics within a marine fish management area
Source: Evol Appl. 2019 Jan 20;12(4):830–44. doi: 10.1111/eva.12760 (PMC6439499; doi:10.1111/eva.12760)
Supplement: Supplementary file 5 [file EVA-12-830-s005.pdf]

Supporting information for: Genetic analyses reveal complex dynamics within a marine fish management area

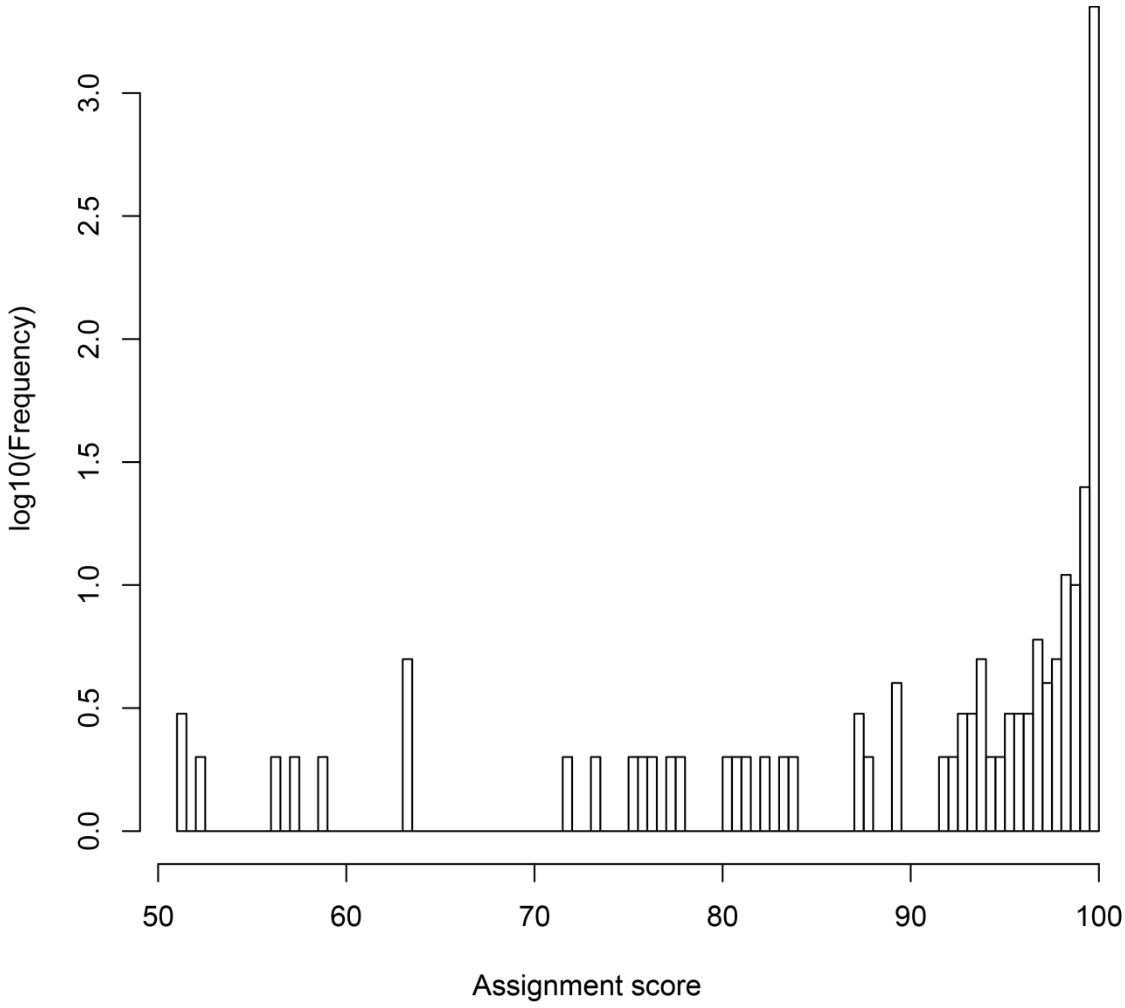

Figure S2. Distribution of assignment scores for individuals analysed with 39 SNP markers. Note that the y axis is log scaled. We added one observation to each assignment score bin to facilitate plotting of observations on a log scale.
