# Supplementary material for: Genetic analyses reveal complex dynamics within a marine fish management area
Source: Evol Appl. 2019 Jan 20;12(4):830–44. doi: 10.1111/eva.12760 (PMC6439499; doi:10.1111/eva.12760)
Supplement: Supplementary file 6 [file EVA-12-830-s006.pdf]

Supporting information for: Genetic analyses reveal complex dynamics within a marine fish management area

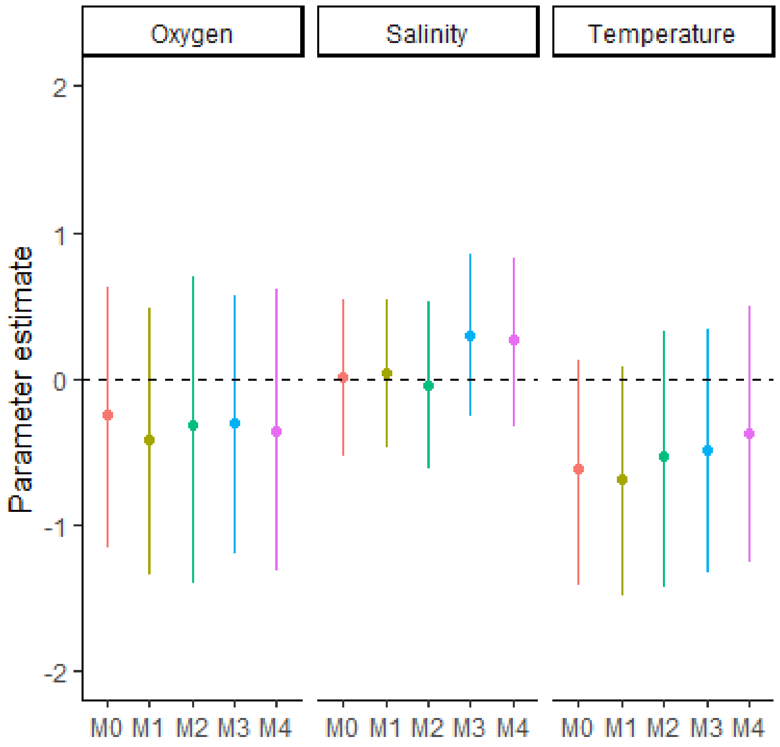

Figure S3. Posterior means and 95 % credible intervals of estimated fixed effects parameters oxygen, salinity and temperature for the five evaluated models.
